# Interleukin-5 levels in relation to malaria severity: a systematic review

**DOI:** 10.1186/s12936-023-04659-3

**Published:** 2023-08-03

**Authors:** Manas Kotepui, Thitinat Duangchan, Aongart Mahittikorn, Chusana Mekhora, Nsoh Godwin Anabire, Kwuntida Uthaisar Kotepui

**Affiliations:** 1 Medical Technology Program, School of Allied Health Sciences, Walailak University, Tha Sala, Nakhon Si Thammarat, Thailand; 2 Department of Protozoology, Faculty of Tropical Medicine, Mahidol University, Bangkok, Thailand; 3 Department of Nutrition and Health, Institute of Food Research and Product Development, Kasetsart University, Bangkok, Thailand; 4 Department of Biochemistry & Molecular Medicine, School of Medicine, University for Development Studies, Tamale, Ghana; 5 Department of Biochemistry, Cell & Molecular Biology, West African Centre for Cell Biology of Infectious Pathogens (WACCBIP), University of Ghana, Accra, Ghana

**Keywords:** Malaria, Severe, Uncomplicated, Complicated, IL-5, Interleukin, Cytokine

## Abstract

**Background:**

The role of cytokines such as interleukin-5 (IL-5) in the pathogenesis of malaria remains unclear. This systematic review sought to synthesize variations in IL-5 levels between severe and uncomplicated malaria, as well as between malaria and controls not afflicted with the disease.

**Methods:**

This systematic review was registered at the International Prospective Register of Systematic Reviews (PROSPERO; CRD42022368773). Searches for studies that reported IL-5 levels in patients with malaria (any severity) and/or uninfected individuals were performed in Web of Science, PubMed, EMBASE, Scopus, CENTRAL, and MEDLINE, between 1st and 10th October, 2022. The risk of bias among all included studies was minimized using the Strengthening the Reporting of Observational Studies in Epidemiology (STROBE) guidelines for reporting observational studies. The differences in IL-5 levels between malaria and uninfected controls, and between severe and uncomplicated malaria were synthesized by narrative synthesis.

**Results:**

Among 1177 articles identified in the databases, 23 matched the eligibility criteria and were included in this systematic review. Qualitative syntheses showed the heterogeneity of IL-5 levels between different severities of clinical malaria and uninfected controls. The majority of the included studies (12/15 studies, 80%) found no change in IL-5 levels between malaria cases and uninfected controls. Similarly, most studies found no difference in IL-5 levels between severe (regardless of complications) and uncomplicated malaria (4/8 studies, 50%). The qualitative syntheses revealed that most studies found no difference in IL-5 levels between severe and non-severe malaria.

**Conclusions:**

The comprehensive review suggests that IL-5 levels are unchanged in patients with different levels of clinical severity of malaria and uninfected controls. Given the limited number of published studies on IL-5 levels in malaria, there is a need for additional research to determine the function of this cytokine in the pathogenesis of malaria.

**Supplementary Information:**

The online version contains supplementary material available at 10.1186/s12936-023-04659-3.

## Background

Malaria in humans is caused by five species of *Plasmodium* parasites: *Plasmodium falciparum*, *Plasmodium vivax*, *Plasmodium ovale*, *Plasmodium malariae*, and *Plasmodium knowlesi*. These parasites are transmitted by the bite of *Anopheles* spp. mosquitoes [1, 2]. Severe malaria is predominantly caused by *P. falciparum*, but other *Plasmodium* species can also cause severe disease, albeit at lower rates [3–6]. Cytokine storm, induced by the infection of *Plasmodium* species, plays a crucial role in the underlying mechanisms of illness and the pathogenesis of malaria [7, 8]. Pro- and anti-inflammatory cytokines have been shown to be involved in systemic inflammation of various diseases including malaria [8]. Previous investigations involving meta-analysis demonstrated that a variety of pro- and anti-inflammatory cytokines, including tumour necrosis factor (TNF), interferon gamma (IFN-γ), interleukin-6 (IL-6), interleukin-4 (IL-4), interleukin-1 (IL-1), interleukin-12 (IL-12), and transforming growth factor beta (TGF-β), are associated with the pathogenesis of malaria [9–15].

Interleukin-5 (IL-5) was first discovered as an interdigitating homodimeric glycoprotein because of its ability to promote the in vitro growth and differentiation of mouse B cells and eosinophils [16]. Eosinophils, mast cells, γδT cells, NK and NKT cells, and non-haematopoietic cells have been proposed as potential immune cells that synthesize IL-5 [17]. IL-5 can enhance expression of the *c-Myc*, *c-Fos*, *c-Jun*, *Cis*, *Cish1/Jab*, and *pim-1* genes in B cells, which induce cell proliferation and have anti-apoptotic effects [18, 19]. In addition, binding of IL-5 to IL-5 receptor (IL-5R) on mouse B cells and eosinophils can activate JAK1/2 and STAT1/5 in the in vitro and in vivo models [20, 21]. In human diseases, it has been proposed that IL-5 plays a role in the pathogenesis of asthma and hypereosinophilic syndromes [17]. Furthermore, modulating the IL-5 pathway holds potential as a therapeutic strategy for treating disorders mediated by eosinophils [22]. Previous studies showed no difference in IL-5 levels between different levels of malaria severity [23–26], the levels of IL-5 in severe malaria anaemia (SMA) and non-SMA [23], or the levels of IL-5 in patients with cerebral malaria and in those with uncomplicated malaria [27]. Given that the role of IL-5 in the pathogenesis of malaria has remained unclear and inconsistent findings were obtained in previous studies, this systematic review was established to synthesize and compare the differences in IL-5 levels between individuals with malaria and uninfected controls, as well as between cases of severe and uncomplicated malaria.

## Methods

### Protocol and registration

This systematic review was registered with the International Prospective Register of Systematic Reviews (PROSPERO; CRD42022368773). This review followed the Preferred Reporting Items for Systematic Reviews and Meta-Analyses protocol (PRISMA 2020 Checklist).

### Search strategy and eligibility criteria

Searches were conducted on six databases, namely, Web of Science, PubMed, EMBASE, Scopus, CENTRAL, and MEDLINE, between 1st and 10th October, 2022. The search strategy was constructed using the keywords “malaria” and “interleukin 5.” A search for synonyms of both keywords was performed using the MeSH terms in the National Center for Biotechnology Information database. The searches were not limited to a particular language or publication date. The details of the search strategy for each database are given in Additional file 1: Table S1. The inclusion criteria for study selection were as follows: (i) the study had a cross-sectional, prospective, or retrospective observational design; and (ii) the study investigated IL-5 levels in patients with malaria (any severity). The following articles were excluded: case reports, case series, letters, news, in vitro studies, reviews, animal studies, studies in which IL-5 was measured after treatment with anti-malarial drugs, and studies from which data on IL-5 could not be extracted.

### Study selection and data extraction

After removing duplicates, two review authors (MK and KUK) independently screened the title and abstract of all of the articles. Then, the full texts of selected studies were examined by the same two authors based on the eligibility criteria. Reasons for the exclusion of any studies were recorded. Any disagreements between the two reviewers were resolved by discussion until a consensus was reached. A third review author (AM) also reviewed and validated the final list of included studies. Two review authors (MK and KUK) independently extracted data from the included studies using a data extraction sheet. Information extracted included name of the first author, study setting (country), year of publication, study period, study design, participant characteristics (e.g., age, sex), IL-5 levels in malaria cases of different severity and uninfected controls, parasite density, method of malaria diagnosis, method of IL-5 quantification, and company/brand of IL-5 reagent. After resolving any disagreements, the extracted data were compared and finalized by the third review author (AM).

### Risk of bias in individual studies

Two review authors (MK and TD) assessed the risk of bias among all included studies using the Strengthening the Reporting of Observational Studies in Epidemiology (STROBE) guidelines for reporting observational studies [28]. This tool assessed the risk of bias among included studies based on 22 items, including title and abstract, background/rationale, objectives, study design, setting, participants, variables, data sources/measurement, bias, study size, quantitative variables, statistical methods, participants, descriptive data, outcome data, main results, and other analyses. Any disagreements between the two review authors were reconciled by reaching a consensus.

### Synthesis of results

The main outcomes of the systematic review included (i) the difference in IL-5 levels between malaria and uninfected controls, and (ii) the difference in IL-5 levels between severe and uncomplicated malaria. Outcome data were synthesized by narrative synthesis as a few studies reported quantitative levels of IL-5 among groups of participants. The significance of difference in IL-5 levels among groups of participants was indicated in the included reports. The percentage of data was calculated using Microsoft Excel 2019 (Version 16.0) (Microsoft Corporation, Redmond, WA, USA).

## Results

### Search results

**Fig. 1:**
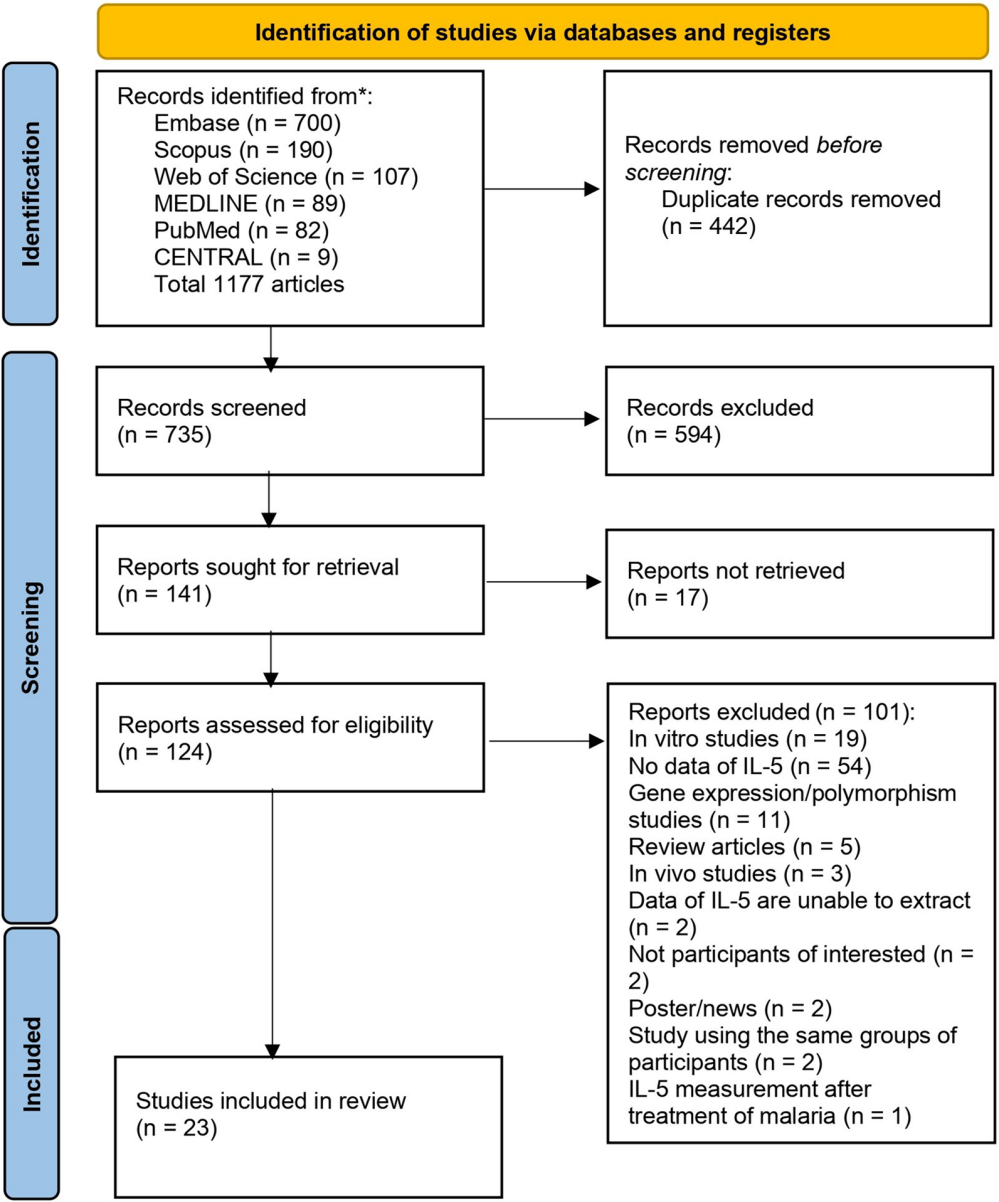
Study flow diagram

A total of 1177 articles were identified from Embase (n = 700), Scopus (n = 190), Web of Science (n = 107), MEDLINE (n = 89), PubMed (n = 82), and CENTRAL (n = 9). After the removal of duplicate records (n = 442), the remaining articles were screened for titles and abstracts (n = 735). After irrelevant records were excluded (n = 594), the remaining articles were sought for retrieval (n = 141), and were assessed for eligibility (n = 124). Twenty-three articles that met the eligibility criteria were included in the systematic review. Meanwhile, 101 articles were excluded for the following reasons: in vitro studies (n = 19), no data on IL-5 (n = 54), gene expression/polymorphism studies (n = 11), review articles (n = 5), in vivo studies (n = 3), inability to extract IL-5 data (n = 2), not participants of interest (n = 2), poster/news (n = 2), study using the same groups of participants (n = 2), and IL-5 measurement was performed after treatment of malaria (n = 1) (Fig. 1).

### Characteristics of the included studies

The 23 studies that were considered eligible for inclusion in this study were all reported between 2000 and 2022, the majority (73.9%) between 2011 and 2022. The largest segment of the included studies employed a cross-sectional design, accounting for 30.4% of the total. This was followed by prospective observational and prospective cohort studies, each comprising 26.1%, and finally case–control studies, which made up 17.4% of the total study designs. The majority of included studies (65.2%) were carried out in Africa, followed by Asia, America, and then Europe (13.0%, 13.0%, and 4.35%, respectively). One study was carried out in five different countries (Brazil, Colombia, Guatemala, India, Papua New Guinea) [29]. Patients with *P. falciparum* infection made up the majority of study participants (82.6%), followed by those with *P. vivax* infection (17.4%). Children (43.5%) made up the majority of the study participants in the included studies, followed by adults (21.7%), pregnant women (17.4%), and both children and adults (17.4%). Most of the included studies used microscopy (69.6%) to detect malaria parasites. The majority of the included studies used plasma (65.2%) and bead-based assays (78.3%) for the quantification of IL-5 levels (Table 1 and Additional file 2: Table S2).

**Table 1:** Characteristics of the included studies

Characteristics	N (23 studies)	%
Year of publication		
2011–2022	17	73.9
2000–2010	6	26.1
Study designs		
Cross-sectional studies	7	30.4
Prospective observational studies	6	26.1
Prospective cohort studies	6	26.1
Case–control studies	4	17.4
Study areas		
Africa	15	65.2
Ghana	5	
Kenya	3	
Tanzania	2	
Benin	2	
Malawi	1	
Mali	1	
Nigeria	1	
Asia	3	13.0
India	3	
America	3	13.0
Brazil	3	
Europe	1	4.35
Spain	1	
More than one country	1	4.35
Brazil, Colombia, Guatemala, India, Papua New Guinea	1	
*Plasmodium* spp.		
*P. falciparum*	19	82.6
*P. vivax*	4	17.4
Participants		
Children	10	43.5
Adults	5	21.7
Pregnant women	4	17.4
Children and adults	4	17.4
Methods for malaria detection		
Microscopy	16	69.6
Microscopy and PCR	3	13.0
Microscopy and RDT and PCR	2	8.70
Microscopy and RDT	1	4.35
Malaria antigen ELISA	1	4.35
Methods for IL-5 quantification		
Bead-based assay	18	78.3
ELISA	5	21.7
Sample for cytokine measurement		
Plasma	15	65.2
Serum	8	34.8

*ELISA* enzyme-linked immunosorbent assay, *PCR* polymerase chain reaction, *RDT* rapid diagnostic test

### Risk of bias in included studies

Among the cross-sectional studies, six were of high quality [23, 30–34], while one was of moderate quality [26]. Among the cohort studies, ten were of high quality [25, 29, 35–42], while two were of moderate quality [43, 44]. Among the case–control studies, three were of high quality [24, 27, 45], while one was of moderate quality [46]. All studies were included for qualitative synthesis (Additional file 3: Table S3).

### Comparative analysis of IL-5 levels in malaria and uninfected controls

Fifteen studies reported IL-5 levels in both malaria and uninfected controls [24, 25, 27, 29, 30, 33–35, 38–41, 43, 45, 46]. Out of these studies, 12 studies demonstrated similar levels of IL-5 between malaria and uninfected controls [24, 25, 27, 29, 30, 33, 35, 39–41, 43, 46]. Chêne et al. reported lower IL-5 levels in malaria patients than in uninfected controls [38], while Da Costa et al. showed that malaria patients had higher IL-5 levels than uninfected controls [45]. Meanwhile, Wilson et al. showed that the TNF:IL-5 ratio differed significantly between the asymptomatic malaria cases and healthy controls [34].

### Differences in IL-5 levels in severe and uncomplicated malaria

Eight studies reported IL-5 levels in severe malaria cases [23–27, 36, 37, 42]. Four studies showed no difference in IL-5 levels between severe malaria (regardless of complications) and uncomplicated malaria [23–26]. One study by Armah et al., which evaluated the levels of IL-5 in severe malaria anaemia (SMA), cerebral malaria, and non-malaria controls, found similar levels of IL-5 across the disease groups [36]. Brickley et al. showed that IL-5 levels were statistically significantly higher in children who developed SMA than in non-SMA cases [37]. One report demonstrated higher levels of IL-5 in patients with cerebral malaria than in those with uncomplicated malaria [27]. Meanwhile, according to a study by Prakash et al., IL-5 levels were markedly elevated in uncomplicated malaria and decreased as the severity of the disease increased (severe non cerebral malaria, cerebral malaria) [42].

### IL-5 levels in malaria co-infections

IL-5 in malaria co-infections was demonstrated by four studies [31, 32, 43, 44]. According to the study by Davenport et al., patients with malaria and bacterial co-infection had significantly higher levels of IL-5 than healthy controls, and the co-infection was also associated with higher levels of IL-5 than in cases of malaria mono-infection [43]. Another study by Davenport et al. showed similar levels of IL-5 in patients with malaria and HIV co-infection, HIV mono-infection, and malaria mono-infection [44]. Moreover, de Oliveira Menezes et al. showed higher IL-5 levels in malaria co-infections with enteroparasites than in endemic controls, and IL-5 levels were higher in malaria co-infection than in malaria mono-infection [31]. Furthermore, Mendonça et al. showed that haemoglobin and haematocrit were negatively associated with IL-5 levels in malaria cases but not in cases of malaria co-infection with dengue [32].

## Discussion

Cytokines have a systemic effect during the immune response of the host to malaria and are gaining popularity in the development of point-of-care diagnostics, not only for malaria but also for other inflammation-mediated diseases [47, 48]. In this systematic review, differences of IL-5 levels between malaria and uninfected controls, and between severe and uncomplicated malaria were synthesized. The qualitative syntheses showed that the majority of included studies found no difference in IL-5 levels between malaria cases and uninfected controls [24, 25, 27, 29, 30, 33, 35, 39–41, 43, 46]. These studies enrolled patients with *P. falciparum*, except for the studies by Dobaño et al. [29] and da Costa et al. [45], which enrolled patients with *P. vivax* malaria. These two studies were conducted in overlapping regions, but they enrolled different participants. Specifically, while Dobaño et al. [29] enrolled pregnant women with asymptomatic malaria, da Costa et al. [45] enrolled adults with uncomplicated malaria. There was homogeneity of the outcome of no difference in IL-5 levels between malaria cases and uninfected controls among the studies that enrolled children [27, 40, 41, 43, 46]. Meanwhile, heterogeneity of IL-5 levels between malaria cases and uninfected controls was observed among pregnant women and adult participants [24, 29, 33–35, 38, 39, 45]. Studies that enrolled pregnant women demonstrated no difference in IL-5 levels between malaria cases and uninfected controls, or showed that the former group had lower IL-5 levels [29, 35, 38]. The heterogeneity of IL-5 levels among these studies may have been caused by the difference in the geographical distribution of *Plasmodium* spp. or the method used for IL-5 measurement. In terms of the methods used to determine cytokine levels, Young et al. suggested that ELISA and bead-based assay yielded similar results for rat IFN-γ, TNF, and IL-6. ELISA was found to be more sensitive in the low range of the standard curve, whereas the bead assay could detect higher protein concentrations [49]. Additionally, bead-based assays can be multiplex, enabling several cytokines to be detected in a platform [50]. Nevertheless, the efficacy of these methods was not compared for IL-5. For this systematic review, 11 studies that used bead-based assays demonstrated homogeneous results of the IL-5 levels between malaria cases and uninfected controls [24, 25, 27, 29, 33, 35, 39–41, 43, 46]. Meanwhile, heterogeneity of IL-5 levels was observed among studies that used ELISA for IL-5 measurement [30, 38, 45].

The qualitative syntheses revealed that most studies found no difference in IL-5 levels between severe and non-severe malaria cases [23–26]. Nonetheless, other studies demonstrated higher or lower IL-5 levels in patients with severe malaria than in those with uncomplicated malaria [27, 42]. In terms of the particular complications, Armah et al. [36] and Mandala et al. [27] showed no difference in IL-5 levels between severe malarial anaemia and cerebral malaria, indicating that IL-5 levels were comparable in groups with complications of different severity. Nevertheless, Brickley et al. showed that IL-5 levels were significantly higher in children with severe anaemia than in those with non-severe anaemia [37]. All of the aforementioned studies that evaluated the difference in IL-5 levels in patients with severe and uncomplicated malaria enrolled patients with *P. falciparum* infection; as such, there was no heterogeneity of *Plasmodium* spp. on the outcome of the synthesis analysis herein. Heterogeneity of IL-5 levels between severe malaria and uncomplicated malaria was observed with respect to geographical area. In Africa, IL-5 levels were either similar or higher in severe malaria cases relative to the levels in uncomplicated malaria [23, 26, 27, 36, 37]. Meanwhile, in Asia similar or lower IL-5 levels were observed in severe malaria cases relative to uncomplicated malaria cases [24, 25, 42]. Regarding the methods used for IL-5 measurement, studies that used bead-based assay showed either similar [23–25] or higher [27, 37] IL-5 levels in severe malaria than in uncomplicated malaria. Studies that used ELISA found similar [26] or lower [42] levels of IL-5 in severe malaria than in uncomplicated malaria.

This systematic review had some limitations. First, there were few studies investigating IL-5 in malaria, so the results of this review were limited. Second, the meta-analysis to pool the mean difference in IL-5 levels among severe malaria, uncomplicated malaria, and uninfected controls could not be performed due to the few studies reporting quantitative data for IL-5 levels between groups of participants.

## Conclusions

This comprehensive review revealed that IL-5 levels did not differ in patients with malaria of various levels of clinical severity and uninfected controls based on the included studies. In the literature, there is only a small number of studies investigating IL-5 levels in malaria; thus, further research is required to determine how this cytokine contributes to the disease’s pathophysiology.

### Supplementary Information

**Additional file 1: Table S1.** Search terms.

**Additional file 2: Table S2.** Details of the included studies.

**Additional file 3: Table S3.** Quality of the included studies. PRISMA abstract checklist. PRISMA 2020 checklist.

## Data Availability

All data relating to the present study are available in this manuscript and Additional files.
