# Supplementary material for: Interleukin-5 levels in relation to malaria severity: a systematic review
Source: Malar J. 2023 Aug 3;22:226. doi: 10.1186/s12936-023-04659-3 (PMC10401852; doi:10.1186/s12936-023-04659-3)
Supplement: Supplementary file 1 — Additional file 1: Table S1. Search terms. [file 12936_2023_4659_MOESM1_ESM.docx]

**Interleukin-5 levels concerning malaria infection and severity: A systematic review**

Manas Kotepui^1^, Thitinat Duangchan^1^, Aongart Mahittikorn^2*^, Chusana Mekhora^3^, Nsoh Godwin Anabire^4^, Kwuntida Uthaisar Kotepui^1*^

^1^ Medical Technology, School of Allied Health Sciences, Walailak University, Tha Sala, Nakhon Si Thammarat, Thailand

^2^Department of Protozoology, Faculty of Tropical Medicine, Mahidol University, Bangkok, Thailand

^3^Institute of Food Research and Product Development. Department of Nutrition and Health, Kasetsart University, Bangkok, Thailand.

^4^Centre for Medical Parasitology, University of Copenhagen, Denmark

**Table S1. Search strategy**

**Embase**

**10 October 2022**

| **No.** | **Query** | **Results** |
| --- | --- | --- |
| #3 | #1 AND #2 | 700 |
| #2 | plasmodium OR 'remittent fever' OR 'marsh fever' OR paludism | 82964 |
| #1 | 'interleukin 5'/exp OR 'interleukin 5' OR 'bcgfii' OR 't-cell-replacing factor'/exp OR 't-cell-replacing factor' OR 't cell replacing factor'/exp OR 't cell replacing factor' OR 'il5'/exp OR 'il 5' OR il5 OR 't-cell replacing factor'/exp OR 't-cell replacing factor' OR 'b-cell growth factor-ii'/exp OR 'b-cell growth factor-ii' OR 'b cell growth factor ii'/exp OR 'b cell growth factor ii' OR 'eosinophil differentiation factor'/exp OR 'eosinophil differentiation factor' | 114493 |

**PubMed**

**10 October 2022**

| **Search number** | **Query** | **Search Details** | **Results** |
| --- | --- | --- | --- |
| 3 | #1 AND #2 | 82 | 3 |
| 2 | malaria[MeSH Terms] | 72,742 | 2 |
| 1 | "Interleukin 5" OR BCGF-II OR "T-Cell-Replacing Factor" OR "T Cell Replacing Factor" OR IL-5 OR IL5 OR "T-Cell Replacing Factor" OR "B-Cell Growth Factor-II" OR "B Cell Growth Factor II" OR "Eosinophil Differentiation Factor" | 18,490 | 1 |

**CENTRAL**

**10 October 2022**

| **No.** | **Query** | **Results** |
| --- | --- | --- |
| #3 | #1 AND #2 | 9 |
| #2 | ((Plasmodium OR “Remittent Fever” OR “Marsh Fever” OR Paludism)):ti,ab,kw | 3202 |
| #1 | (“Interleukin 5” OR BCGF-II OR “T-Cell-Replacing Factor” OR “T Cell Replacing Factor” OR IL-5 OR IL5 OR “T-Cell Replacing Factor” OR “B-Cell Growth Factor-II” OR “B Cell Growth Factor II” OR “Eosinophil Differentiation Factor”):ti,ab,kw | 1262 |

**Scopus**

**10 October 2022**

| **Databases** | **Search terms/Search strategy** |
| --- | --- |
| Scopus | (TITLE-ABS-KEY ( "interleukin 5" OR bcgf-ii OR "t-cell-replacing factor" OR "t cell replacing factor" OR il-5 OR il5 OR "t-cell replacing factor" OR "b-cell growth factor-ii" OR "b cell growth factor ii" OR "eosinophil differentiation factor" ) ) AND ( TITLE-ABS-KEY ( plasmodium OR "remittent fever" OR "marsh fever" OR paludism ) )  Search results: 190 |

**MEDLINE**

**10 October 2022**

| **Databases** | **Search terms/Search strategy** |
| --- | --- |
| MEDLINE | (“Interleukin 5” OR BCGF-II OR “T-Cell-Replacing Factor” OR “T Cell Replacing Factor” OR IL-5 OR IL5 OR “T-Cell Replacing Factor” OR “B-Cell Growth Factor-II” OR “B Cell Growth Factor II” OR “Eosinophil Differentiation Factor”) AND (Plasmodium OR “Remittent Fever” OR “Marsh Fever” OR Paludism)  Search results: 89 |

**Web of Science**

**10 October 2022**

| **Databases** | **Search terms/Search strategy** |
| --- | --- |
| **Web of Science** | (“Interleukin 5” OR BCGF-II OR “T-Cell-Replacing Factor” OR “T Cell Replacing Factor” OR IL-5 OR IL5 OR “T-Cell Replacing Factor” OR “B-Cell Growth Factor-II” OR “B Cell Growth Factor II” OR “Eosinophil Differentiation Factor”) AND (Plasmodium OR “Remittent Fever” OR “Marsh Fever” OR Paludism)  Search results: 107 |
